# Masquelet technique combined with a lantern-mimicking frame system for treatment of a 28-cm infected bone defect: a case report

**DOI:** 10.3389/fsurg.2025.1577774

**Published:** 2025-04-29

**Authors:** Ji Qv, Feng Gu, Yanbing Wang, Chuangang Peng, Dankai Wu

**Affiliations:** Department of Orthopedics, 2nd Hospital of Jilin University, Changchun, China

**Keywords:** longest bone defect, bone infection, case report, Masquelet technique, lantern-mimicking frame system

## Abstract

**Introduction:**

Treatment of large bone defects resulting from acute injury or infection remains challenging. The Masquelet technique is a two-stage procedure for treating bone defects caused by bone tumor resection, infection, or trauma. There are currently no reports of successful repair of ≥28 cm bone defects using the Masquelet technique.

**Case presentation:**

We describe the case of a 55-year-old man with postoperative infection of a femoral fracture and a 28-cm infected bone defect formed after multiple debridement procedures. The Masquelet technique, when coupled with a Lantern-Mimicking Frame System (LMFS), achieved favorable clinical results. The patient could walk normally without crutches 14 months postoperatively and did not experience pain in daily life.

**Conclusions:**

This was the longest bone defect in a single limb currently reported to had been cured in the literature. The Masquelet technique coupled with LMFS achieved favorable clinical results for the treatment of a 28-cm infected bone defect. For extremely large bone defects in a single limb, the length of the defect was not an absolute limiting condition for the indications of Masquelet technique.

## Introduction

Osteomyelitis is a serious complication of orthopedic surgery that can cause large bone defects. The Masquelet technique was first described by Masquelet et al. (1, 2) to treat bone defects caused by bone tumor resection, infections, or trauma. It is a two-stage procedure. In the first stage, a polymethyl methacrylate (PMMA) bone-cement spacer is placed in the defect to induce a foreign body reaction, resulting in induced membrane formation. In the second stage, the spacer is removed from the defect site and replaced with a bone graft (3). Cierny et al. (4) classified osteomyelitis as types A, B, and C based on physiological considerations and types I–IV based on anatomic considerations.

Many studies have shown that bacteria can affect normal tissues surrounding infected areas and that local antibiotics and irrigation may not effectively control such infections (5). Therefore, in addition to tissue infections, debridement of normal tissues is required during therapy. Although we hope to minimize surgical trauma, complete bone removal and radical debridement are necessary when repeated debridement fails to control infection. However, this often leads to large bone defects and poor clinical outcomes. Repair of large bone defects has always been challenging. This is the first report of a 28-cm bone defect treated using the Masquelet technique; currently, it is the longest segment bone defect treated with the technique. In the present case, a novel Lantern-Mimicking Frame System (LMFS) was used, and clinical outcomes remained favorable after more than 25 months of follow-up.

## Case presentation

**Figure 1:**
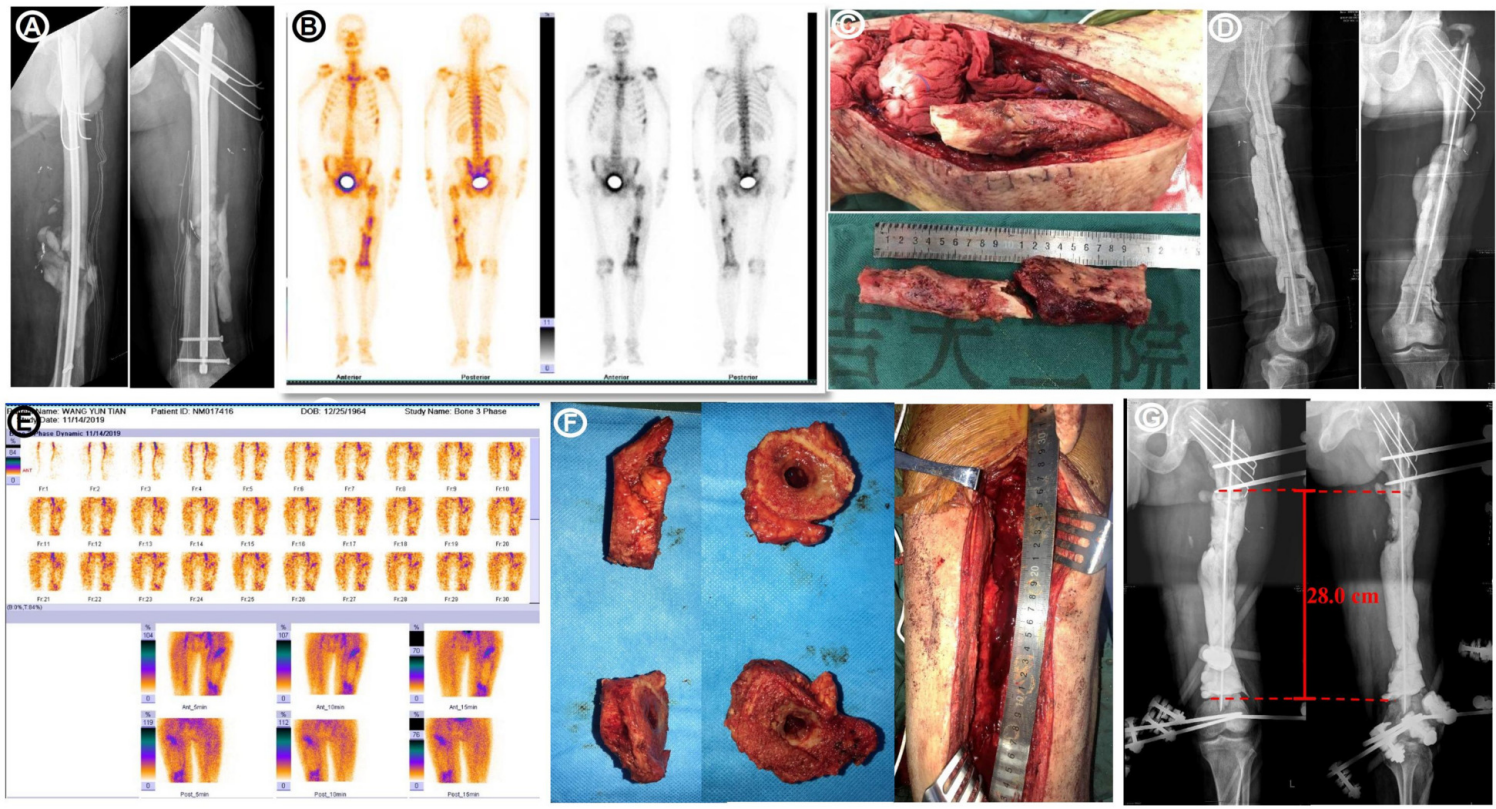
Images of the first and second surgeries. **(A)** Radiographs obtained before the first debridement. **(B)** SPECT/CT results showed positive blood flow, pooling, and delayed phases. **(C)** A large infection at the fracture end was observed during the second debridement and surgical resection of 20 cm of the infected bone. **(D)** Post-resection and antibiotic-loaded PMMA rod imaging. **(E)** Before the third debridement, SPECT/CT results indicated positive blood flow, pool, and delay phases. **(F)** Surgical resection of 8 cm of the infected bone. **(G)** Radiographs obtained after second debridement.

A 55-year-old man experienced closed left intertrochanteric and left femoral shaft fractures due to a traffic accident. He had no history of diabetes or immunodeficiency. He underwent open reduction and internal fixation with intramedullary nails at a local hospital and developed an infection 2 months after surgery. At a local hospital he underwent debridement, but the infection remained uncontrolled. He visited our hospital because of a red and swollen incision accompanied by a wave sensation experienced 3 months after debridement. The surgical incision was obviously red and swollen on admission, and the palpation skin temperature increased and tenderness was obvious. There was no fluctuation and sinus formation, so no bacterial culture was carried out on admission. The patient's knee and hip joints were limited due to pain. At admission, his hemoglobin was 98 g/L, serum C-reactive protein level was 111.0 mg/L, and the erythrocyte sedimentation rate was 104.0 mm. Single-photon emission computed tomography (SPECT)/computed tomography (CT) showed that the blood flow, blood pool, and delayed phases were positive, with the thickening of the adjacent soft tissue shadow indicating infection (Figures 1A,B).

### Surgical procedure

**Figure 2:**
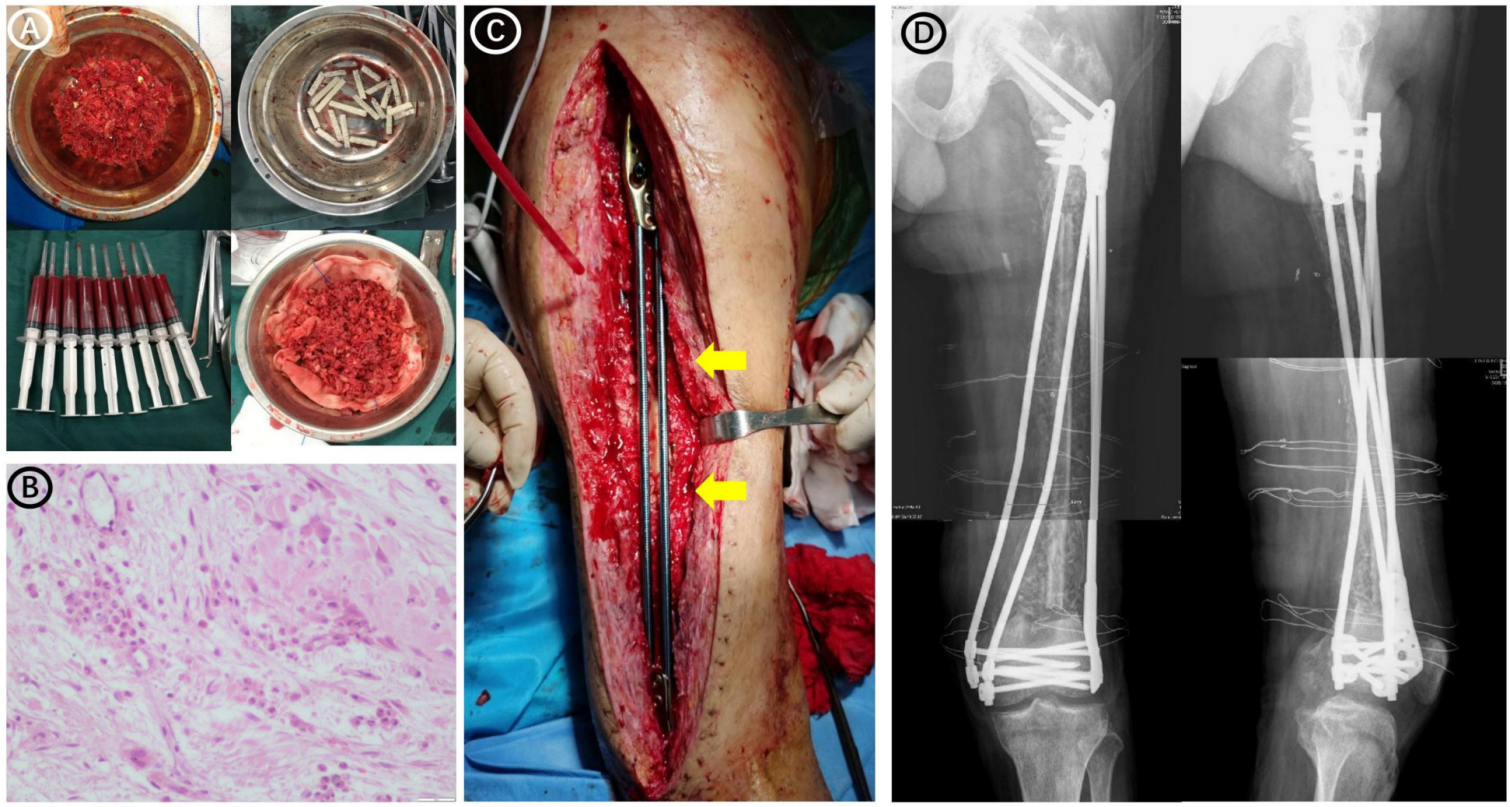
Preoperative and postoperative images of the patient undergoing a bone graft. **(A)** Autologous iliac bone, autologous bone marrow stem cells, and bone morphogenetic protein-loaded artificial bone used in surgery. **(B)** Pathological examination during bone grafting surgery revealed a few inflammatory cells infiltrating the soft tissue around the bone defect, and the number of neutrophils was less than 5 in each high-power (×400 magnification) field of view. **(C)** Intraoperative image showing the bridging internal fixation (yellow arrows). **(D)** Radiographs obtained after bone grafting and bridging of the internal fixation.

**Figure 3:**
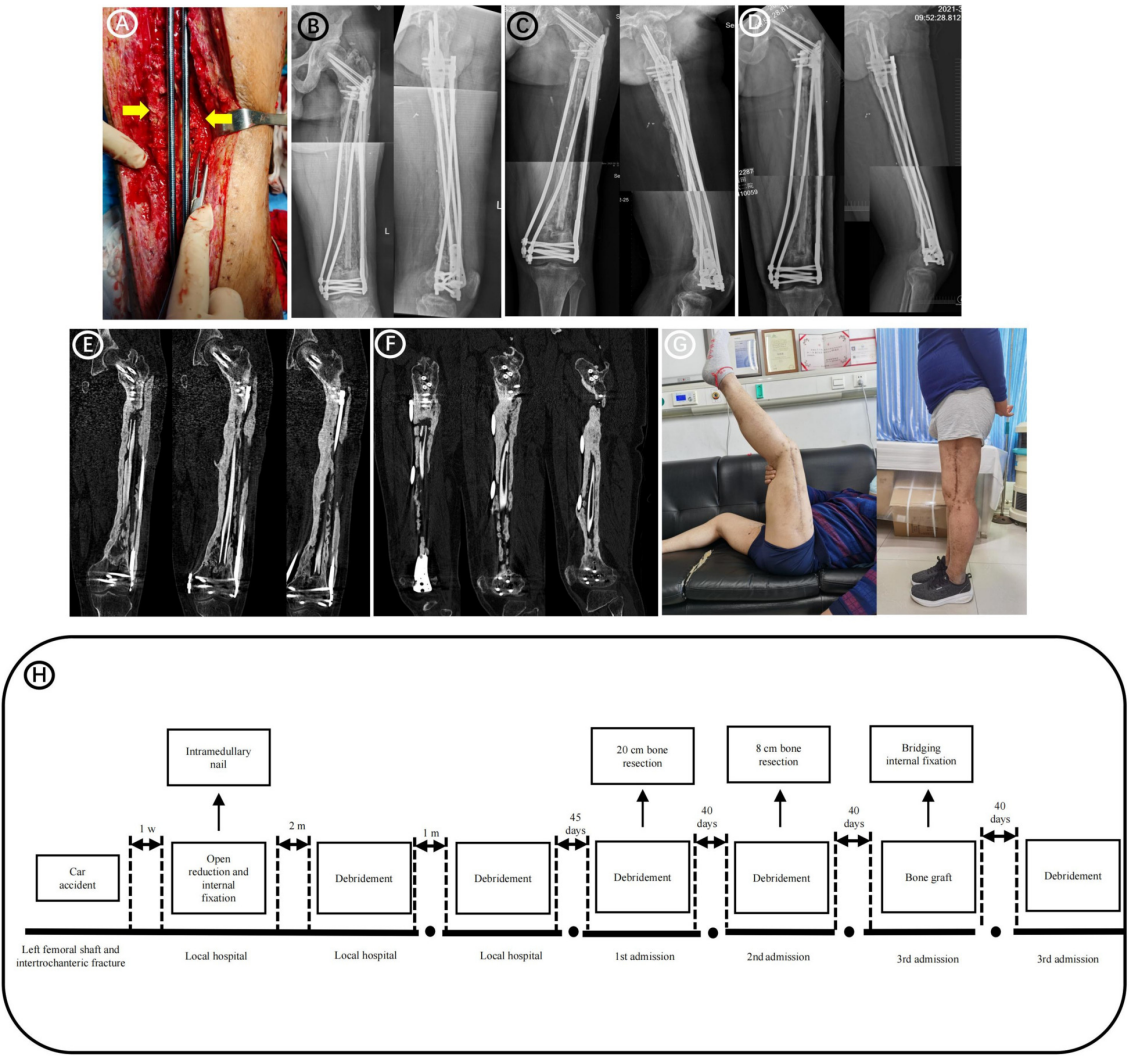
Follow-up images after bone grafting surgery. **(A)** Forty days after bone grafting surgery, debridement surgery and antibiotic-loaded PMMA implantation were performed due to the high long-term drainage volume of the incision. During the debridement surgery it was observed that all bones in the previous bone-grafting area were adhered to the induction membrane, which provided blood supply (yellow arrows). **(B)** Radiographs obtained 4 months after bone grafting. **(C)** Radiographs obtained 8 months after bone grafting. **(D)** Radiographs obtained 14 months after bone grafting. **(E)** Postoperative CT 25 months after bone grafting (Coronary CT). **(F)** Postoperative CT 25 months after bone grafting (Sagittal CT). **(G)** Postoperative functional imaging 14 months after bone grafting. **(H)** The patient's treatment timeline.

The infected area was thoroughly cleaned, and bone destruction and extensive infection of the surrounding soft tissues were evident. Twenty centimeters of the femur were cut off, and the implant was removed. Bacterial cultures were collected at three points during the operation, and the results were negative. The defect was filled with an antibiotic PMMA rod (Heraeus Medical GmbH, Wehrheim, Germany) and an elastic intramedullary nail (Figures 1C,D). 2 g vancomycin (Vancocin, Italia S.R.L.) and 2 g imipenem (Merck Sharp & Dohme Corp.) were mixed into every 40 g PMMA. After one month, the patient was hospitalized again, and his serum C-reactive protein was 11.1 mg/L and the erythrocyte sedimentation rate was 7.0 mm. SPECT/CT revealed positive blood flow, blood pools, and delayed phases (Figure 1E). A second debridement was performed, and bone destruction was still evident at the fractured end of the femur (Figure 1F); therefore, a total of 8 cm of bone was removed from both ends (Figure 1F). At this point the total length of the femoral defect reached 28 cm (Figure 1F), and the femur was fixed using an external fixator (Figure 1G). Bacterial cultures were collected at three points during the debridement, and the results were negative. White blood cells (WBCs), C-reactive protein (CRP), and erythrocyte sedimentation rate (ESR) were checked every 2 weeks. Only after they were normal for more than two consecutive times could the stage II operation be performed. Forty days after the third debridement procedure, the patient was admitted to our hospital for bone grafting. The external fixator, elastic intramedullary nail, and PMMA were removed, and bilateral autogenous iliac bone, allogeneic fibula, autologous iliac bone, autologous bone marrow stem cells, and bone morphogenetic protein-loaded artificial bone were used to perform sufficient bone grafting. Due to the huge length of bone defect, the remaining bone was not suitable for fixation with plate or intramedullary nail, while the external fixation frame was difficult to provide sufficient stability. Therefore, using Ortho-Bridge System (OBS, Tianjin Weiman Biomaterials Co., Ltd.), a novel LMFS method designed by our team was applied to bone reconstruction (Figures 2A–D). This lantern structure could not occupy the bone graft space of bone defect during the bone graft stage, and the blood supply of the induced membrane was protected to the greatest extent. The OBS passed through the muscle tissue or subcutaneously according to the situation, and the two ends of femur were fixed stereoscopically. The two OBSs on the medial side were minimally invasive. Forty days after the bone grafting surgery, due to high long-term drainage volume of the incision, debridement surgery and antibiotic PMMA implantation were performed. Few studies have reported relationships between the induced membrane and the transplanted bone. In the present case, during debridement surgery it was observed that all the bones in the previous bone grafting area were adhered to the induction membrane, which provided blood supply (Figure 3A). Bacterial cultures were collected at three points during the debridement, and the results were negative.

### Postoperative care

The patient was not allowed to bear weight on the affected limb, and limited functional exercises were performed on the bed until an imaging examination showed that the bone had healed. Throughout his hospital stay, he was administered intravenous antibiotics and rivaroxaban.

### Outcomes

Postoperative radiographs showed a good line of force and an adequate amount of bone graft in the bone defect area. Follow-up results showed that the femur had healed well 14 months after bone grafting (Figures 3B–F). The patient could walk normally without crutches at 14 months postoperatively, with no pain in daily life (Figure 3G). His American Orthopedic Foot-and-Ankle Society score was 88, and his Hospital for Special Surgery score was 86. Figure 3H shows the entire treatment timeline.

## Discussion

The treatment of large bone defects resulting from acute injury or infection remains challenging (6). Autologous bone grafting is the gold standard for treating bone defects (7). A critical-sized bone defect is defined as one that is too wide to heal spontaneously, or with a standard cancellous bone graft (8). The Masquelet technique and distraction osteogenesis (Ilizarov technique) have recently been used to treat critical-sized bone defects. When distraction osteogenesis is used to treat large bone defects, the treatment can require a prolonged time in an external fixator, with some series reporting up to 2 months per centimeter of bone defect (9). The extensive process of distraction osteogenesis carries significant risks for large bone defects measuring 28 cm. Therefore, we believe that the Masquelet technology was a better choice for the current patient. We reviewed 13 recent studies involving 389 patients, in which the longest bone defect treated using the Masquelet technique was 17.4 ± 5.5 cm (Table 1). Herein, we describe the application of the Masquelet technique combined with an OBS to treat a 28-cm infected bone defect, the longest segment thus treated reported to date.

**Table 1:** Bone defect characteristics of previous studies.

Articles	Number of cases	Maximum length of bone defect (cm)	First-stage fixation	Second-stage fixation
Apard (22)	12	15	Nail	Nail
Azi (23)	33	15.5	External fixation	Nail/plate
Donegan (24)	11	15	Nail/plate	External fixation/plate
El Alfy (25)	17	11	External fixation	External fixation
Karger (26)	84	23	External fixation/nail/plate	External fixation/nail/plate
Masquelet (27)	31	25	External fixation/nail/plate	External fixation/nail/plate/wires
Moghaddam (28)	50	26	External fixation/nail/plate	External fixation/nail/plate
Orbert (29)	9	12	Nail	Nail
Scholz (30)	13	14.5	External fixation/nail/plate	External fixation/nail/plate
Stafford (31)	27	25	Nail/plate	Nail/plate/plate + nail
Taylor (32)	69	14	External fixation/NAIL/PLAte	External fixation/nail/plate
Wang (33)	32	12.5	External fixation external fixation + nail/plate	External fixation/external fixation + nail/plate/plate + nail
Kyle Kubes (34)	1	20	Nail	Nail

Thorough debridement is the basis for the treatment of bone infection. Bone reconstruction under the premise of controlling infection is the principle for the treatment of bone infection. Preconditions for implant retention include a stable osteosynthetic construct, a vital soft tissue envelope, the ability to perform proper debridement, and a time interval between fracture fixation and infection manifestation (10). In principle, removal of internal fixation is considered necessary when infection occurs after intramedullary nail implantation surgery (10, 11). Confirming the required debridement range is challenging when treating bone infections. Elevated serum C-reactive protein levels and erythrocyte sedimentation rate before surgery suggest the possibility of infection. The presence of neutrophils in biopsy samples (>5 PMNs/HPF) is included in the FRI consensus definition as a confirmatory criterion (12). Nuclear imaging helps determine the extent of infection. Despite its high diagnostic accuracy, nuclear imaging is still not a conclusive test for establishing the presence of FRI and is, therefore, categorized as suggestive in the consensus definition (10, 12). Simpson et al. (13) reported good clinical outcomes after 3–5-mm marginal resection for type A and 5-mm marginal resection for type B. During debridement, we observed that the infection had spread to the end of the fracture and caused extensive bone destruction. Bone resection was therefore performed, which resulted in the formation of a final 28-cm bone defect. The continuous presence of large drainage volume 40 days after operation does not exclude the presence of infection, but may also be related to the excessive bone defect and the presence of local lacunae after bone grafting. Anyway, if infection cannot be excluded, we believe that timely debridement is necessary, and the symptoms of patients have been controlled through debridement.

Another challenge was the timing and method of bone grafting during the second stage of the Masquelet technique. Some studies suggest that osteogenic and neovascular activity in the membranes is maximal between 2 and 4 weeks and subsides after 6 weeks (14); however, these conclusions were based on animal experiments and do not fully represent human clinical practice. Clinical studies have suggested that prolonging the time of bone grafting in the induction membrane does not affect final bone healing (15). In the current patient, the infection could not be controlled within a short period; therefore, bone grafting was performed 4 months after bone cement implantation, and the patient ultimately achieved bone healing. The most common way of bone transplantation is the autologous iliac bone, and others include vascularized fibula transplantation, Reamer-Irrigator-Aspirator (RIA), rib transplantation and so on. Masquelet et al. suggested additional augmentation with allografting or a demineralized bone substitute at a ratio of ≤1:3 (autograft: allograft) to achieve sufficient graft volume or strength (16).

Fracture stability is crucial for bone consolidation and infection eradication. Experimentally contaminated fractures without internal fixation were more prone to infection than fractures treated with internal fixation (17, 18). Another key point was the choice of the fixation method. In the first stage, external fixation was performed to fix the fracture. Fixation in the second-stage surgery was very difficult because the external fixator could not provide sufficient strength for definitive fixation. Common internal fixation methods include locking compression plate (LCP) and intramedullary nail. The advantage of intramedullary nails is that they can be placed minimally and allow for dynamization in some cases to promote bone healing. However, once infection occurs in intramedullary fixation, it may lead to disastrous consequences. LCP can provide more stable mechanical strength, but these two kinds of internal fixation are not suitable for use when the bone volume at both ends of the bone defect is too small. Hence, an OBS was used to fix the proximal and distal femoral ends. Wang et al. (19) described this system, which, as a clamp-locking internal fixation system, has the advantages of external fixation, locking plates, and intramedullary nails. Some studies have used OBSs to treat ipsilateral proximal and femoral shaft fractures and achieved good results (20). Other studies have reported that the system has certain advantages for the treatment of periprosthetic fractures (21). A novel LMFS method designed by our team was applied to bone reconstruction. It is a stereoscopic fixation method, and the interior bridging fixation is minimally invasive. In the present case, internal fixation was performed outside of the inducing membrane without interfering with the osteogenic induction area. We believe that this induced external fixation method is very important for fracture healing because it reduces the damage and disturbance of the blood supply to the bone graft area.

## Conclusions

This was the longest bone defect in a single limb currently reported to had been cured in the literature. In the present case, an infected 28-cm bone defect was successfully treated, which has never been reported in the literature. The Masquelet technique combined with bridging internal fixation achieved good clinical results. For extremely large bone defects in a single limb, the length of the defect was not an absolute limiting condition for the indications of Masquelet technique.

## Data Availability

The original contributions presented in the study are included in the article/Supplementary Material, further inquiries can be directed to the corresponding authors.
